# Genetics, Molecular Mechanisms, and Prevention of Rubber Tree (*Hevea brasiliensis*) Anthracnose in China

**DOI:** 10.3390/plants15203105

**Published:** 2026-10-10

**Authors:** Zhiying Cai, Liming Dai, Lili He, Yuping Shi, Yixian Liu, Haipeng Su

**Affiliations:** Tropical Crop Diseases Research Group, Research Center of Plant Protection, Yunnan Key Laboratory of Sustainable Utilization Research on Rubber Tree, Yunnan Institute of Tropical Crops, Jinghong 666100, China

**Keywords:** *Hevea brasiliensis*, anthracnose, epidemiology, control, molecular mechanism, integrated disease management

## Abstract

Rubber tree (*Hevea brasiliensis*) anthracnose, caused by multiple species within the genus *Colletotrichum*, is a globally significant disease that seriously threatens the sustainable development of the natural rubber industry in China. Characteristic symptoms, including foliar necrosis, defoliation, and shoot blight, culminate in significant economic losses for producers. This review systematically summarizes the epidemiological patterns and damage, pathogen genetic diversity, the molecular pathological mechanisms underlying *Colletotrichum*–rubber tree interactions, and chemical and biological control strategies. Furthermore, an integrated disease management (IDM) framework is proposed, and key directions for future research are outlined to provide a theoretical foundation for the scientific prevention and control of rubber tree anthracnose in China.

## 1. Introduction

Natural rubber is an indispensable industrial and strategic raw material, predominantly sourced from the para rubber tree, *Hevea brasiliensis* Muell. Arg. [1,2]. As a perennial tropical crop, rubber production is persistently constrained by diverse biotic stresses, among which anthracnose ranks as one of the most destructive foliar disease complexes [3]. Rubber tree anthracnose is a complex disease caused by a diverse and evolving group of *Colletotrichum* species, with *Colletotrichum gloeosporioides* and *Colletotrichum siamense* being the predominant causal agents in major rubber-growing regions of China [4,5]. This disease can occur throughout the entire growth period of rubber trees, especially affecting young leaves, shoots, inflorescences, and fruits in nurseries, as well as virgin trees and mature tapping trees in rubber plantations. Over the past decade, advances in control and prevention technologies and molecular tools have substantially accelerated the discovery of key components in the rubber tree defense system. Effective management requires an integrated disease management (IDM) approach that combines cultural practices to reduce humidity and stress, the strategic use of effective chemical fungicides, and rapid molecular diagnostic tools such as the loop-mediated isothermal amplification (LAMP) assay [6]. Fundamental research has begun to unravel the molecular mechanisms, identifying key fungal pathogenicity factors, such as CsAtf1 and basic leucine zipper (bZIP) transcription factors [7,8,9], the microtubule end-binding protein-encoding gene *CgEB1* in *C. gloeosporioides,* and host signaling pathways that govern the infection process and plant resistance [10]. In this review, we searched English and Chinese literature on rubber tree anthracnose in PubMed (https://pubmed.ncbi.nlm.nih.gov/) (accessed on 1 June 2026) and the China National Knowledge Infrastructure (CNKI, https://www.cnki.net/) (accessed on 1 June 2026) databases for the period from 2000 to 2026, with keywords including ‘rubber tree anthracnose’, ‘*Colletotrichum*’, and ‘*Hevea anthracnose*’, and screened original studies and reviews related to epidemiology, etiology, and control strategies. This review consolidates recent findings on the epidemiological patterns and damage, pathogen genetic diversity, the molecular mechanisms underlying *Colletotrichum*–rubber tree interactions, and chemical and biological control strategies in *H. brasiliensis*. Future research should focus on leveraging this molecular knowledge for the development of novel, durable, and sustainable control strategies, including advanced molecular diagnostics, resistant clones through marker-assisted selection, and new biological control agents.

## 2. Epidemiological Patterns and Damage of Rubber Tree Anthracnose

The epidemic of anthracnose is closely related to meteorological conditions, host phenology, and cultivation management [11]. The disease exhibits peak severity under high-temperature and high-humidity conditions. In Hainan province, the rainy season from May to July provides optimal conditions for pathogen infection and reproduction. Vulnerable host tissues, such as tender young leaves, are particularly susceptible during their emergence. Furthermore, the pathogen overwinters on diseased plant debris, serving as the primary inoculum source for the subsequent year. In addition, cultivation measures such as excessive planting density, dense canopy coverage, and excessive nitrogen fertilizer application, which promotes elongated young leaves, can all exacerbate disease occurrence.

### 2.1. Epidemic History of Rubber Tree Anthracnose

In 1905, the occurrence of rubber tree anthracnose was first reported in Sri Lanka. Subsequently, cases were documented in rubber plantations across Malaysia, India, Thailand, and China, establishing it as a common disease in rubber-growing regions. In China, the first records of rubber tree anthracnose were limited to sporadic occurrences in nurseries and newly planted seedlings, classifying it as a minor disease. In 1962, at Dafeng Farm in Hainan province, the disease caused leaf drop and prevented latex harvesting from individual tree clones, marking the first recorded case in China. In 1992, a widespread outbreak occurred at Changhao Farm in Hainan province, affecting 75% of the harvested area, with over 200,000 trees exhibiting severe leaf drop as Grade 4–5 (Table 1), leading to delayed harvesting and losses of 250 tons of dry rubber content (DRC) that year. Due to extensive replanting and promotion of high-yield clones, the disease has become increasingly severe. In 1996, affected areas in Hainan reached 730,000 hectares, resulting in losses of 15,000 tons of DRC. In 2004, varying degrees of old-leaf anthracnose and winter-spring new-branch anthracnose were observed in rubber plantations in Xishuangbanna, Honghe, Pu’er, Lincang, and Wenshan City in Yunnan province, causing significant leaf loss and reduced yields, elevating the disease from a minor to a major threat. Between 2012 and 2013, rubber tree anthracnose typically occurred during the young-leaf stage under high-humidity conditions, with two to three peak infection periods annually and severity increasing each year in Hekou City, Yunnan province. In recent years, rubber tree anthracnose and rubber tree powdery mildew have been collectively recognized as the two major leaf diseases affecting rubber trees in China. The incidence of these diseases continues to rise, establishing them as two of the most prominent leaf diseases affecting rubber trees in China.

### 2.2. Damage Caused by Rubber Tree Anthracnose

**Figure 1:**
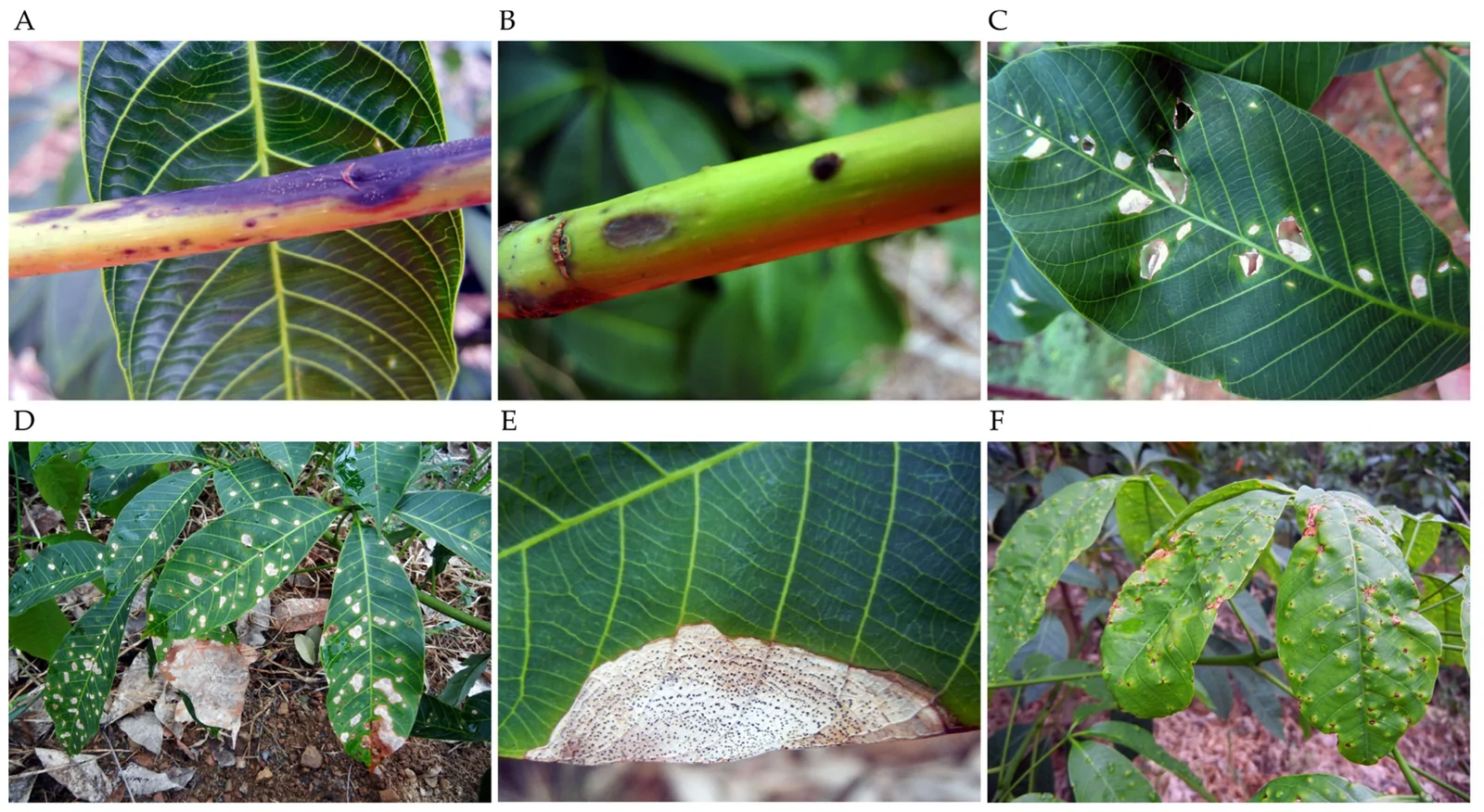
The symptoms of rubber tree (*Hevea brasiliensis*) anthracnose. (**A**), Dark, depressed lesion on the petiole; (**B**), Dark, depressed hole on the petiole; (**C**), Irregular hole on the mature leaflet; (**D**), Cone-shaped protrusions; (**E**), Dead spots on the leaf margin; (**F**), Round or irregular necrotic lesions on light-green leaflets.

Rubber tree anthracnose can affect seedlings in nurseries, virgin trees in plantations, and even mature tapping trees, infecting vulnerable parts such as young leaves, petioles, and young shoots [12]. This disease causes leaf drop and shoot withering. In severe cases, it leads to recurrent leaf loss and shoot withering, delaying the tapping season. Symptoms vary depending on the affected tissue. For instance, both young and mature leaves are susceptible. Bronze-colored young leaves initially develop irregular dark green water-soaked lesions at the tips and margins, which progress to blackening, necrosis, twisting, and eventual leaf detachment and branch drying. Light-green young leaves exhibit circular or irregular lesions at the tips and margins, featuring large dark green water-soaked spots resembling scalded surfaces, sometimes with black necrotic areas at edges. Under high humidity, pink viscous spore clusters often form on infected tissues. In light-green or senescent leaves, multiple patterns emerge, including circular and semicircular types. Initial small water-soaked spots expand into semicircular lesions at tips/margins or circular lesions across leaves. In the irregular type, early lesions appear gray-brown or reddish-brown, with distinct boundaries between diseased and healthy tissue; later lesions merge into irregular patches with perforations. The lesions form small convex cones with wrinkled margins; in severe cases, the entire leaf surface may be covered with upward-convex spots accompanied by perforations. Anthracnose often manifests as small black dots on mature leaves, which may appear arranged in whorls or scattered randomly. Infected young shoots, petioles, and veins exhibit black depressed spots or black streaks. Affected young shoots not only shed leaves but also develop terminal bud necrosis (Figure 1).

In epidemic years, severe leaf drop can cause newly tapped rubber trees to stop tapping, significantly reducing rubber yields. Additionally, during the nursery stage, this disease can cause seedling death; in mature trees, it weakens their vigor, reduces their cold and drought resistance, and shortens their economic lifespan. Current management relies heavily on fungicide applications. Therefore, clarifying its occurrence patterns and prevention techniques is crucial for ensuring the safety of the natural rubber industry. However, growing concerns regarding environmental safety, fungicide resistance, and economic costs underscore the necessity of developing host resistance through molecular breeding. Clarifying the molecular basis of resistance is therefore a prerequisite for efficient and targeted breeding programs.

## 3. Historical Identification of Pathogens in the Anthracnose Complex in China

Rubber tree anthracnose is not caused by a single pathogen but by a complex of species within the genus *Colletotrichum*. Early anthracnose fungi were classified and named based on their host range or the microscopic morphological characteristics of pathogenic vegetative forms on hosts. The taxonomy of *Colletotrichum* species causing rubber tree anthracnose has been a subject of ongoing debate and revision. In 1905, Petch from Sri Lanka first reported rubber tree anthracnose and identified the pathogens as *Gloeosporium heveae* Petch, *Gloeosporioides heveae* Petch, and *Gloeosporioides alborubrum* Petch according to their host range and the presence of conidia and sporangia on hosts. The Fujian rubber tree anthracnose pathogen was named *Colletotrichum heveae* Petch in the China Fungal Compendium, whereas the Yunnan rubber tree pathogen was named *G. alborubrum*. Identification based solely on host range often led to homotypic names for many species. Subsequently, using morphological characteristics of pure cultures of anthracnose fungi (conidia, sporangia, appressoria, etc.) and host range, the anamorph of the rubber anthracnose pathogen was identified as *C. gloeosporioides* f. *heveae* Penz., while the teleomorph was identified as *Glomerella cingulata*. Therefore, prior to 2008, the primary pathogens responsible for rubber tree anthracnose in China were identified as either *C. heveae* or *C. gloeosporioides*.

There are three taxonomic restrictions. The first concerns morphology versus molecular systematics. Identification relying predominantly on morphological features often made it difficult to distinguish between closely related species. Early studies suggested that anthracnose in rubber trees was mainly caused by *C. gloeosporioides* and *Colletotrichum acutatum* in China. With advancements in molecular biology, the internal transcribed spacer (ITS) region of ribosomal DNA has been widely utilized in fungal species identification. Researchers have increasingly employed a combination of morphological identification and molecular analysis methods for identifying rubber tree anthracnose pathogens. The identification now typically integrates morphological characteristics with ITS single-gene sequence analysis. In 2008, researchers identified additional pathogens besides *C. gloeosporioides* causing anthracnose in rubber trees in Xishuangbanna, Yunnan, through observation of conidial morphology, including *C. acutatum*. Subsequently, the isolation of *C. acutatum* from rubber trees in Hainan, Guangdong, and Guangxi was independently confirmed using ITS-rDNA sequences. In 2014, the authors designed specific primers based on ITS sequences to conduct molecular identification and distribution analysis of *Colletotrichum* in China. Results showed that among 138 isolates collected from Hainan, Yunnan, Guangdong, and Guangxi, 72.46% were identified as *C. gloeosporioides*. Thus, between 2008 and 2012, two species, *C. gloeosporioides and C. acutatum,* were identified as causative agents of rubber tree anthracnose in China, with *C. gloeosporioides* being the predominant species in field populations. However, ITS sequence analysis still exhibits limitations in taxonomic studies of anthracnose fungi, particularly in accurately reflecting or distinguishing the phylogenetic relationships among closely related species (Table 2).

The second taxonomic restriction is species complex delimitation. The multigene classification method has been widely adopted and recognized for the identification of fungal species. Currently, the identification of anthracnose primarily relies on polygenic sequence assembly analysis combined with morphological observation. Within the *C. gloeosporioides* complex, two species are predominant: *C. siamense* and *Colletotrichum fructicola*, with *C. siamense* being the dominant species in field populations [13]. The adoption of multilocus phylogenetic analysis (using 5–8 gene regions) has fundamentally revised our understanding of *Colletotrichum* taxonomy. What was previously considered a single species, *C. gloeosporioides*, is now recognized as a complex of multiple phylogenetically distinct but morphologically similar species. Researchers demonstrated that three species, *C. siamense*, *C. fructicola*, and a new species, *Colletotrichum wanningense*, are associated with rubber tree anthracnose in Hainan [15]. Similarly, studies in Yunnan revealed nine species, including two novel species, *Colletotrichum jinpingense* sp. nov. and *Colletotrichum mengdingense* sp. nov. Sreenivasaprasad [17]. These findings indicate that the species diversity of rubber tree anthracnose pathogens is still underestimated and that continued taxonomic revision is necessary.

The third taxonomic restriction is the species concept debate. There is an ongoing debate within the *Colletotrichum* research community regarding the relative weighting of morphological, phylogenetic, and biological species concepts. Some researchers advocate for a pragmatic approach using genealogical concordance phylogenetic species recognition (GCPSR) [18], while others emphasize the need for complementary evidence from mating-type distribution, host range, and pathogenicity assays to define biologically meaningful species boundaries (Table 3).

## 4. Molecular Mechanisms of Host–Pathogen Interactions

**Figure 2:**
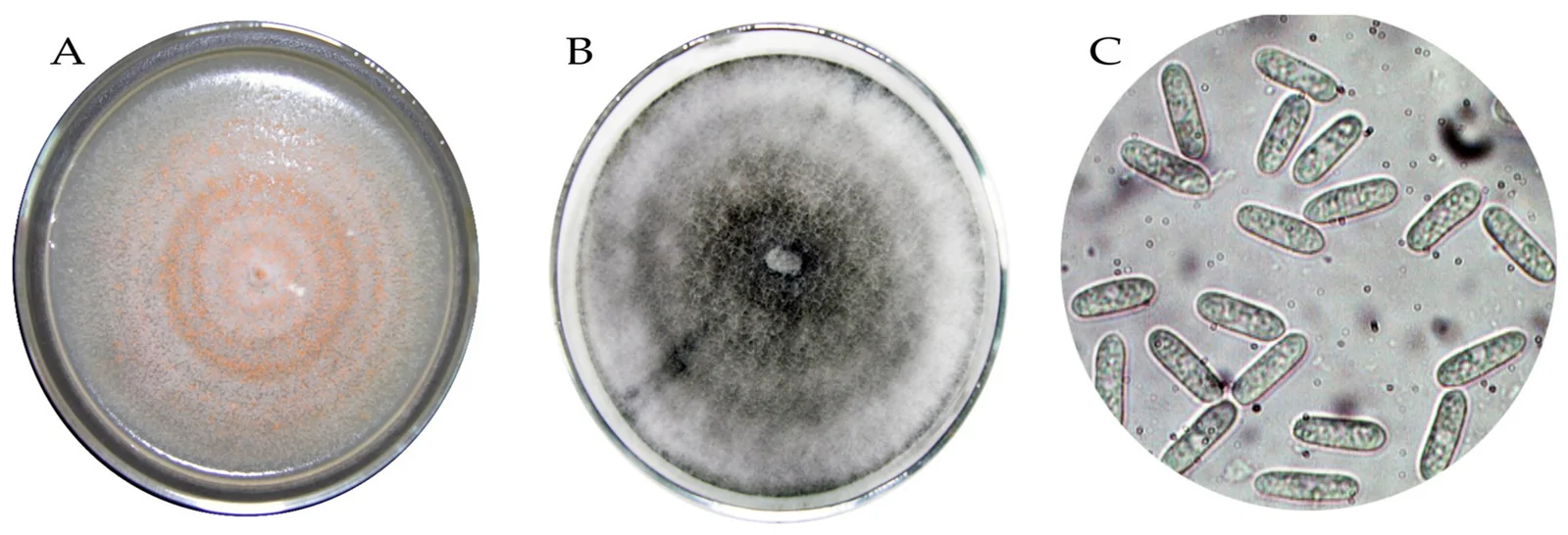
Colony morphology and spore structure of the rubber anthracnose fungus (*Colletotrichum* species). (**A**) Colored conidia; (**B**) Dark gray conidia; (**C**) Microscopic view of conidial structures.

Figure 2 collectively illustrates the colony morphology and spore structure of the rubber tree anthracnose fungus (*Colletotrichum* sp.), aiding in its identification and studies of infection. The successful infection by pathogens depends on a series of complex pathogenic factors. Necrosis- and ethylene-inducing peptide 1-like proteins (NLPs) from *Colletotrichum australisinense,* the causal agent of rubber tree anthracnose, exhibit necrosis-inducing activity [22]. The G-protein alpha subunit CgGa1 mediates growth, sporulation, penetration, and pathogenicity in *C. gloeosporioides* [23]. The study found that the bZIP transcription factor CsAtf1 in *C. siamense* plays a key role in regulating the pathogen’s sensitivity to the fungicide fludioxonil and its pathogenicity. Knockout of *CsAtf1* affected spore germination and appressorium formation of the pathogen, thereby weakening its pathogenicity to rubber trees [7]. Actin-bundling protein fimbrin regulates pathogenicity via organizing F-actin dynamics during appressorium development in *C. gloeosporioides* [24]. The effector protein CgNLP1 of *C. gloeosporioides* affects invasion and disrupts nuclear localization of necrosis-induced transcription factor hbmyb8-like to suppress plant defense signaling [25]. The fatty acid 2-hydroxylase CsSCS7 is a key hyphal growth factor and potential control target in *C. siamense* [26].

### 4.1. Pathogen Virulence Factors and Signaling Pathways

#### 4.1.1. The CsAtf1 Transcription Factor and HOG MAPK Pathway

The high-osmolarity glycerol (HOG) mitogen-activated protein kinase (MAPK) pathway plays a critical role in stress response and pathogenicity in *C. siamense*. The transcription factor CsAtf1, belonging to the ATF/CREB-type bZIP transcription factor family, functions downstream of the HOG MAPK pathway [7]. CsAtf1 participates in the response to fludioxonil stress, with Δ*CsAtf1* mutants exhibiting resistance levels similar to those of *Hog1* and *Pbs2* deletion mutants (Table 4). Through ChIP-seq and RNA-seq analyses, 3809 significantly differentially expressed genes were identified as directly regulated by CsAtf1, including 24 cytochrome oxidase-related genes.

#### 4.1.2. The CsAtf1–CsCyp51G1 Regulatory Module

One of the cytochrome P450 genes targeted by CsAtf1, designated *CsCyp51G1*, was confirmed as a direct target using electrophoretic mobility shift assay (EMSA), yeast one-hybrid (Y1H), and real-time quantitative reverse transcription PCR (qRT-PCR) assays. The transcriptional expression of *CsCyp51G1* is negatively regulated by CsAtf1. *CsCyp51G1* overexpression mutants showed increased tolerance to fludioxonil, while *CsCyp51G1* deletion mutants showed decreased resistance, indicating that *CsCyp51G1* participates in regulating fludioxonil sensitivity in *C. siamense*. Interestingly, the ergosterol content in mutants was inconsistent with the fludioxonil sensitivity phenotype, suggesting that *CsCyp51G1* regulates fludioxonil sensitivity through mechanisms beyond ergosterol levels. This establishes a regulatory module in which the HOG MAPK pathway and transcription factor CsAtf1 negatively regulate *CsCyp51G1* gene expression, thereby increasing fludioxonil sensitivity in *C. siamense* [8].

#### 4.1.3. Melanin Biosynthesis and Pathogenicity

Melanin is a complex secondary metabolite critical to fungal pathogenicity, contributing to mechanical penetration by generating high turgor pressure within appressoria, scavenging reactive oxygen species, and strengthening the fungal cell wall. In *C. siamense*, melanin biosynthesis is essential for the formation of melanized appressoria and primary hyphae. Transcriptomic analysis revealed that two major melanin biosynthesis pathways, the DHN (1,8-dihydroxynaphthalene)-melanin pathway and the DOPA (3,4-dihydroxyphenylalanine)-melanin pathway, are active in *C. siamense* [27].

The velvet proteins CsVosA and CsVelB coordinate growth, cell wall integrity, sporulation, conidial viability, and pathogenicity in the rubber anthracnose fungus *C. siamense* [28]. The CgATG16 protein is involved in growth, development, and virulence through autophagy modulation in the rubber tree anthracnose fungus *C. gloeosporioides* [29]. The Cg2LysM effector protein has been confirmed to be produced by *C. gloeosporioides*. It enhances virulence to rubber trees by affecting the invasive structure of appressoria and inhibiting plant-triggered immune responses by binding to chitin [30]. The biotrophy-associated secreted protein (BAS2) is crucial for the biotrophic phase of the pathogen. Although it does not affect mycelial growth, it is essential for conidiogenesis and pathogenicity and is secreted into host cells during infection [31]. Additional characterized pathogenicity factors in *Colletotrichum* species infecting rubber trees include CgEB1 (microtubule end-binding protein regulating infection-related morphogenesis) [32], CgNLP1 (Nep1-like protein cytolytic toxin inducing host cell death) [25], CgMFS1 (major facilitator superfamily transporter for efflux of fungicides and host antimicrobial compounds) [33], CgNPG1 (non-ribosomal peptide synthetase for siderophore biosynthesis) [27], CsSCS7 (serine palmitoyltransferase for sphingolipid biosynthesis) [26], and the CgAzf1 transcription factor (C_2_H_2_ zinc-finger protein regulating melanin production, conidial development, and infection) [34].

**Table 4:** Reported pathogenicity/virulence factors of the *Colletotrichum* species complex on rubber trees.

Gene Name	Species	Encoded Protein	Biological Function	Key References
*CsAtf1*	*Colletotrichum siamense*	bZIP transcription factor	Regulates fludioxonil sensitivity and virulence; affects spore germination and appressorium formation	[7,8]
*CgEB1*	*Colletotrichum gloeosporioides*	Microtubule end-binding protein	Regulates vegetative growth, conidiation, appressorium formation, and pathogenicity; involved in salt stress response	[10]
*CsSCS7*	*Colletotrichum siamense*	Fatty acid 2-hydroxylase	Key hyphal growth factor; potential control target	[26]
*CsVosA/CsVelB*	*Colletotrichum siamense*	Velvet domain proteins	Coordinate growth, cell wall integrity, sporulation, conidial viability, and pathogenicity	[28]
*CgATG16*	*Colletotrichum gloeosporioides*	Autophagy-related protein	Involved in growth, development, and virulence through autophagy modulation	[29]
*Cg2LysM*	*Colletotrichum gloeosporioides*	LysM effector protein	Binds chitin to inhibit plant-triggered immune responses; affects appressorium invasive structure	[30]
*BAS2*	*Colletotrichum gloeosporioides*	Secreted protein	Essential for conidiogenesis and biotrophic phase pathogenicity; secreted into host cells during infection	[31]
*CgNPG1*	*Colletotrichum gloeosporioides*	Novel pathogenic gene	Regulates mycelial growth, conidiation, and invasive structure development	[27]
*CgNLP1*	*Colletotrichum gloeosporioides*	Nep1-like protein	Affects invasion and disrupts nuclear localization of necrosis-induced transcription factor HbMYB8-like to suppress plant defense signaling	[25]
Fimbrin	*Colletotrichum gloeosporioides*	Actin-bundling protein	Regulates pathogenicity via organizing F-actin dynamics during appressorium development	[24]

### 4.2. Host Defense Mechanisms

#### 4.2.1. Plant Probiotic Traits of Biocontrol-Associated Microorganisms

*Bacillus velezensis* SF334 harbors several plant probiotic traits that contribute to disease suppression, including the secretion of siderophores, protease, cellulase, pectinase, and the auxin indole-3-acetic acid (IAA). It also exhibits broad-spectrum antifungal activity against important plant pathogenic fungi such as *C. siamense*, *C. australisinense*, *Magnaporthe oryzae*, *Fusarium oxysporum*, *Alternaria solani*, and *Botrytis cinerea* [35].

#### 4.2.2. Defense Enzyme Activation

Treatment with biocontrol agents such as *Bacillus altitudinis* WR7 activates defense-related enzymes in rubber tree leaves, including catalase (CAT) and superoxide dismutase (SOD), thereby enhancing the plant’s defense responses against pathogen invasion [3]. Studies on chilling stress responses in rubber tree clones have demonstrated that activation of the antioxidant enzyme system is an important factor determining stress tolerance. In the chilling-tolerant clone GT1, SOD, peroxidase (POD), and CAT activities were significantly higher than in the cold-sensitive clone Wenchang217 under low-temperature conditions, while malondialdehyde (MDA) content was significantly lower, indicating reduced lipid peroxidation [20].

Recent research has also identified SNARE (soluble N-ethylmaleimide-sensitive factor attachment protein receptor) proteins as key regulators of anthracnose resistance in rubber trees. A total of 117 SNARE genes were identified within the rubber tree genome. Among them, HbSNAP33 and HbSYP4 were highlighted as key regulators, showing strong induction during pathogen infection. Functional analysis revealed that these genes enhance anthracnose resistance by modulating the salicylic acid (SA) signaling pathway and stabilizing reactive oxygen species (ROS) homeostasis. Notably, HbSNAP33 activated not only SA signaling but also jasmonic acid (JA) signaling, pattern-triggered immunity (PTI), and effector-triggered immunity (ETI), while HbSYP4 predominantly enhanced SA-associated basal defense [36,37,38,39].

#### 4.2.3. Pathogenesis-Related (PR) Proteins and Long Non-Coding RNAs

A novel PR-10 protein (LOC110648447) was identified in rubber tree leaves in response to pathogen infection. The promoter of this PR-10 gene contains WRKY, MYB, and other defense-related cis-acting elements, and its structural model showed a topology of the Bet v 1 protein family, including a conserved active site and a ligand-binding hydrophobic cavity, suggesting a potential role in defending rubber trees against pathogen infection [40]. The functional roles of HblncRNA29219 and its antisense hbr-miR482a, as well as the miR390-TAS3-ARF pathway, in enhancing anthracnose resistance have been reported [41,42].

### 4.3. Host Specificity and Host Shift Mechanisms

The ability of *Colletotrichum* species to infect and cause disease on *H. brasiliensis* involves a complex interplay of pathogen virulence factors and host susceptibility determinants. Several mechanisms facilitate host shifts and the expansion of the host range in *Colletotrichum*. The first is effector protein evolution and diversification. The *Colletotrichum* genome harbors large repertoires of candidate secreted effector proteins (CSEPs) that evolve rapidly under positive selection. Host shifts often involve the gain, loss, or modification of effector genes that allow the pathogen to evade or suppress the immune system of a new host. The second is metabolic adaptation to the host environment. Successful infection requires the pathogen to adapt to the nutritional and chemical environment of the host tissue. *Colletotrichum* species possess extensive suites of cell wall-degrading enzymes (CWDEs), including cutinases, pectinases, cellulases, and hemicellulases, that must be compatible with the specific polysaccharide composition of rubber tree cell walls. The third is temperature and environmental adaptation. Different *Colletotrichum* species and isolates vary in their temperature optima for growth and infection. *C. acutatum* species complex members generally have lower temperature optima (20–25 °C) than *C. gloeosporioides* complex members (25–30 °C) [2]. This differential temperature adaptation may facilitate host shifts under changing climatic conditions and partly explains the geographic distribution patterns of different species across China’s rubber-growing regions. The fourth is genetic recombination and hybridization. Whole-genome resequencing has revealed genetic recombination phenomena within the *C. acutatum* species complex [20]. Sexual reproduction and parasexual recombination can generate new genotypic combinations with altered host ranges or increased aggressiveness.

## 5. Disease Prevention and Control Techniques

### 5.1. Chemical Control

Chemical control remains the primary method for managing anthracnose in rubber plantations. The key to successful chemical control depends on selecting critical application times, such as when 30% of rubber trees have defoliated, especially when weather forecasts predict continuous rain or heavy fog. Several fungicide classes have demonstrated effective control against rubber tree anthracnose in China. The first group comprises strobilurins (e.g., azoxystrobin and pyraclostrobin). These fungicides inhibit mitochondrial respiration by binding to the Qo site of cytochrome bc1 (Complex III), blocking electron transfer and ATP synthesis in fungal cells. They also exhibit translaminar movement, providing some curative activity. The second group comprises triazoles (e.g., difenoconazole, tebuconazole, and propiconazole). These sterol demethylation inhibitors (DMIs) target C14-demethylase (CYP51) in the ergosterol biosynthesis pathway, disrupting fungal cell membrane integrity. The third group comprises dithiocarbamates (e.g., mancozeb). These multi-site contact fungicides disrupt multiple thiol-containing enzymes in fungal cells through metal ion chelation and SH-group inactivation, making resistance development less likely. The fourth group comprises protectant fungicides, including chlorothalonil and copper-based formulations. These broad-spectrum fungicides interfere with fungal enzyme systems through non-specific protein denaturation and glutathione depletion. In field trials, 40.44 g/L fenhexamid and tebuconazole hot mist showed excellent control efficacies of 70.80% and 76.73%, respectively, 30–60 days after application. 10% chlorothalonil thermal spray and 20% fluosilazole thermal spray achieved average efficacies of 68.14% and 66.31%, respectively, with thermal spraying proving 3–4 times more effective than conventional ground-based spraying. 28% compound carbendazim rubber suspension, applied when 5% to 80% of rubber trees show sprouting or bronze-colored young leaves, achieved an average control efficacy of 74.5% under appropriate timing [43].

### 5.2. Special Strategies for Leaf Fall Control

Some studies have explored methods to control *Colletotrichum* leaf drop disease (CLD) through artificial leaf drop. In the GT1 clone, this method significantly increased leaf density and rubber yield by approximately 40%, and by inducing early leaf drop, new leaves can avoid fungal attack, effectively preventing disease outbreaks. Numerous studies have reported the evaluation of the efficacy of common pesticides against rubber anthracnose using the colony growth rate method in vitro. Endemic fungicides such as imidacloprid, carbendazim, and thiophanate-methyl exhibit higher antifungal efficacy compared to protective fungicides like zinc pyroximate and tebuconazole.

It was demonstrated that artificial leaf fall using defoliants can effectively control rubber leaf anthracnose, promoting uniform leaf shedding in rubber trees and consistent leaf emergence, thereby avoiding peak disease incidence periods and increasing rubber yield [44]. Researchers in Malaysia reported excellent efficacy of chlorothalonil oil for controlling anthracnose pathogens. Application of artificial defoliants such as dimethasone or sodium dimethasone promotes leaf fall, mitigating adverse growth conditions and reducing pathogen damage. Chinese researchers employed a 28% compound carbendazim rubber suspension during the period when 5% to 80% of rubber trees in forest stands exhibited sprouting or bronze-colored young leaves. If low-temperature rainy weather was anticipated, the first application should be made 2–3 days prior to onset. If low-temperature rainy conditions were forecasted 7–10 days post-application, a second or third application could achieve an average control efficacy of 74.5%. Studies have found that treatment of bronze-stage rubber leaves with BTH (benzothiazole) significantly reduced anthracnose severity compared to controls, indicating BTH-induced systemic resistance in rubber plants. In 2008, we utilized 10% chlorothalonil thermal spray and 20% fluosilazole thermal spray for anthracnose control, achieving average efficacies of 68.14% and 66.31%, respectively; thermal spraying proved 3–4 times more effective than conventional ground-based spraying.

The young rubber leaves represent the most susceptible stage for anthracnose infection. Therefore, disease prediction and early warning systems should be strengthened during this period to enable timely chemical control measures. Most rubber plantations in Yunnan are located in mountainous areas, making large-scale mechanized spraying difficult. Thermal aerosol formulations are ideal for controlling mountain rubber anthracnose, as they are not restricted by water sources or terrain conditions, saving both labor and time. However, attention must be paid to application timing and weather conditions to achieve optimal control efficacy.

### 5.3. Agricultural Control and Variety Resistance

Agricultural control is fundamental to the control of rubber tree anthracnose. This includes improving cultivation and management to enhance tree vigor; timely pruning of diseased branches to improve ventilation and light penetration; and cleaning up diseased leaves and other debris to reduce sources of pathogens. Breeding and planting disease-resistant clones are the most economical and effective strategy. Regarding the resistance of rubber tree clones, research findings vary among scholars due to differences in cultivated clones across regions. Evaluations of different rubber tree clones, such as IAC 40 and IAC 301, have shown that these clones exhibit high resistance to anthracnose [45]. Field disease resistance evaluation of Chinese rubber tree germplasm also screened for excellent anthracnose-resistant germplasm. In the 1980s, the resistance of 34 widely cultivated rubber clones was assessed to anthracnose disease through nursery identification and field surveys. Among these clones, Reyan 44-9, Baoting 933, and Nanqiang 1-97 exhibited resistance to anthracnose. B143, Nanhua 1, PB86, and Reyan 10-14-38 were highly susceptible. Resistant clones demonstrated longer incubation periods, delayed spore production, lower spore yields, and lower disease severity after artificial inoculation, whereas susceptible clones showed short incubation periods, early spore production, high spore yields, and severe disease severity. In vitro inoculations on isolated leaves revealed that the incubation period and disease severity index were strongly correlated with variety resistance, making this method suitable for early identification of rubber variety resistance (Table 5).

Current methods for evaluating the disease resistance of rubber tree clones include field surveys, artificial inoculation assessments under greenhouse conditions, and the in vitro leaf bud method. However, due to varying phenological periods among different clones, these field and greenhouse evaluation methods often yield limited results within a short timeframe. The in vitro approach, controlling temperature and humidity while performing artificial inoculation on isolated young leaf buds and assessing disease resistance using a severity index, is a rational and practical method for evaluating rubber tree variety disease resistance.

### 5.4. Biological Control and Molecular Detection

Biological control represents an important direction for future green disease management. The use of biopesticides, including antagonistic microorganisms and plant defense inducers, has gradually gained attention. In disease monitoring, molecular detection technology has made significant progress. A rapid detection method based on loop-mediated isothermal amplification (LAMP) was established, capable of specifically and sensitively detecting the rubber tree anthracnose complex. It is suitable for detecting pathogens during the latent and symptomatic phases because it is simple and rapid and does not require expensive equipment, providing a new tool for early diagnosis and prevention [6]. Similarly, a real-time PCR method based on the internal transcribed spacer (ITS) has been developed to specifically detect and quantify *C. siamense* and plays important roles in early disease diagnosis and epidemiological research [48].

### 5.5. Integrated Disease Management (IDM)

An integrated disease management (IDM) approach is essential for sustainable control of rubber tree anthracnose, combining chemical, biological, agricultural, and molecular diagnostic strategies.

#### 5.5.1. Cultural Practices

Regular pruning to improve canopy ventilation and reduce within-canopy humidity minimizes conditions favorable for pathogen infection. Optimal planting density management prevents excessive canopy closure. Balanced fertilization—avoiding excessive nitrogen application—prevents the formation of succulent young leaf tissues that are highly susceptible to infection. Balanced potassium and calcium nutrition enhances cell wall integrity and host defense responses. Removal and destruction of infected plant debris during and after the growing season reduces primary inoculum levels for the following year. Intercropping with non-host species can reduce disease pressure by modifying the microclimate and interrupting pathogen dispersal.

#### 5.5.2. Chemical Control Strategy

Alternating between fungicides with different modes of action, e.g., strobilurins, triazoles, multi-site protectants, delays the development of resistance in pathogen populations. Spray timing should target the critical window of young leaf emergence (bronze to light-green stage), particularly when weather forecasts predict three or more days of rain, continuous drizzle, or heavy fog. Fungicide application is triggered when 5% to 30% of trees show new leaf emergence under favorable infection conditions [4]. Weather-based disease forecasting systems that integrate temperature, relative humidity, rainfall, and leaf wetness duration data can optimize spray timing and reduce unnecessary applications. For rubber plantations in mountainous areas (especially in Yunnan), thermal aerosol formulations are recommended, as they are not restricted by water sources or terrain conditions, saving both labor and time.

#### 5.5.3. Biological Control Integration

Biological control agents should be integrated into IDM programs primarily as preventive treatments during the early leaf emergence stages. Bacillus and Streptomyces formulations can be applied as soil drenches or foliar sprays at 7–14-day intervals during high-risk periods. In an IDM context, biocontrol agents can be alternated with reduced-rate fungicides to maintain efficacy while minimizing chemical inputs and selection pressure for resistance.

#### 5.5.4. Molecular Diagnostics for Early Detection

Rapid and accurate pathogen identification at the species level is critical for targeted management, as different *Colletotrichum* species may vary in their fungicide sensitivity, optimal temperature for infection, and aggressiveness on different rubber tree clones. LAMP assays enable rapid, field-deployable detection of *Colletotrichum* species, allowing growers to implement control measures before disease outbreaks reach economic thresholds.

#### 5.5.5. Host Resistance Deployment

The deployment of resistant rubber tree clones through marker-assisted selection (MAS) represents the most sustainable long-term component of IDM. Although no anthracnose-specific resistance genes have been cloned from rubber tree to date, ongoing genomic selection (GS) approaches using multi-environment models and kernel methods have demonstrated superior predictive accuracy for complex traits. Promising resistant germplasm (e.g., clones IAC 40 and IAC 301) should be prioritized for incorporation into breeding programs.

## 6. Conclusions and Future Perspectives

Despite significant progress in the study of anthracnose in rubber trees, many challenges remain. Future research should focus on the following areas. Continuous monitoring of the dynamic evolution of anthracnose pathogens in different rubber-growing areas, particularly the distribution, epidemic potential, and pathogenic differentiation of emerging species such as *Colletotrichum cliviae*, provides a basis for formulating long-term control strategies [5]. In-depth investigation of the molecular interaction mechanisms between pathogen effector proteins, such as Cg2LysM and host resistance proteins [30], has revealed gene-for-gene interaction patterns, providing a theoretical foundation for the design of broad-spectrum disease resistance genes [30] to traditional antagonistic bacteria. Research and field application of plant immune inducers, such as oligosaccharides and RNA interference (RNAi) technologies, warrant greater attention to facilitate the development of environmentally friendly control products. By integrating rapid detection technologies such as LAMP and qPCR with the Internet of Things (IoT), big data, and artificial intelligence, a precise intelligent early-warning model based on meteorological, phenological, and pathogen-monitoring data can be established to enable real-time disease prediction and targeted prevention and control. New technologies such as genome-wide association analysis (GWAS), virus-induced gene silencing (VIGS) [49] and gene editing (e.g., clustered regularly interspaced short palindromic repeats-associated protein 9 [CRISPR/Cas9]) can be employed to rapidly identify and validate key genetic determinants of anthracnose resistance in rubber trees, thereby accelerating the breeding of superior resistant clones. Future research should also systematically characterize the effector repertoire of key *Colletotrichum* strains and identify their cognate nucleotide-binding leucine-rich repeat (NLR) receptors in rubber tree to define specific resistance gene–effector pairs. Expanding genomic resources and validated molecular markers should be leveraged to expedite the development and deployment of high-yielding, anthracnose-resistant rubber tree clones through marker-assisted and genomic selection [50]. By bridging the gap between molecular discovery and practical application, insights derived from the rubber tree immune system will be instrumental in fostering a more resilient and sustainable natural rubber industry.

Despite these advances, several limitations must be addressed to translate research findings into practical disease management. First, long breeding cycles (25–30 years) for rubber tree cultivars remain a major bottleneck for developing resistant varieties through conventional breeding. Second, low genetic transformation efficiency in *Hevea brasiliensis* limits the application of genetic engineering and genome editing approaches. Third, limited genomic resources for non-model resistance donors (wild *Hevea* species) restrict the introgression of novel resistance alleles. Fourth, field efficacy of biocontrol agents under diverse environmental conditions requires extensive validation before commercial deployment. Furthermore, fungicide resistance management strategies need continuous updating as pathogen populations evolve under selection pressure. Finally, diagnostic capacity in remote rubber-growing areas needs strengthening for rapid, species-level pathogen identification.

## Figures and Tables

**Table 1:** Rubber tree (*Hevea brasiliensis*) anthracnose disease grade classification standards based on the percentage of spot area to total leaf area.

Disease Grade	Percentage of Spot Area to Total Leaf Area
0	No anthracnose spots on the leaf
1	≤1/16
2	≤1/8
3	≤1/4
4	≤1/2
5	≤3/4

**Table 2:** Diagnostic methods for rubber tree anthracnose pathogens.

Method	Target Gene/Region	Specificity	Sensitivity	Detection Time	Advantages	Limitations	Key References
Morphological identification	—	Low (species complex)	Low	3–7 days	Simple, no specialized equipment	Cannot distinguish closely related species; requires sporulation	[5,13]
ITS sequencing	ITS1-5.8S-ITS2 rDNA	Moderate	Moderate	1–2 days	Universal primers, widely used	Limited resolution within species complexes; cannot separate *C. siamense*/*C. fructicola* reliably	[5,14]
Multi-gene phylogeny	ITS + ACT + CAL + CHS-1 + GAPDH + TUB2 + HIS3	High (species-level)	High	2–3 days	Gold standard for species identification	Time-consuming; requires multiple PCR reactions and sequencing	[4,15,16]
Species-specific PCR	Species-specific loci	High	High	3–4 h	Rapid, cost-effective	Requires prior knowledge of target species	[6]
LAMP assay	Species-specific target	High	Very high (10–100× PCR)	30–60 min	Rapid, on-site detection; isothermal; visible to the naked eye	Requires careful primer design; risk of carryover contamination	[6]
Real-time qPCR	Species-specific target	High	Very high	1–2 h	Quantifiable; high throughput	Requires expensive equipment and trained personnel	[6]

**Table 3:** Colletotrichum species associated with rubber tree anthracnose in China.

Species	Species Complex	Geographic Distribution in China	Year First Reported on Rubber in China	Key References
*Colletotrichum siamense*	*Colletotrichum gloeosporioides* complex	Hainan, Yunnan, Guangdong, Guangxi	2008 (dominant species)	[5,15,19]
*Colletotrichum fructicola*	*Colletotrichum gloeosporioides* complex	Hainan, Yunnan	2014	[4,15]
*Colletotrichum australisinense*	*Colletotrichum acutatum* complex	Hainan, Yunnan	2014	[19,20]
*Colletotrichum wanningense*	*Colletotrichum acutatum* complex	Hainan	2014	[15]
*Colletotrichum bannaense*	*Colletotrichum acutatum* complex	Yunnan (Xishuangbanna)	2014	[16]
*Colletotrichum cliviae*	*Colletotrichum cliviae* clade	Hainan (Qiongzhong)	2014	[5]
*Colletotrichum brevisporum*	*Colletotrichum brevisporum* clade	Yunnan	2021	[21]
*Colletotrichum plurivorum*	*Colletotrichum orchidearum* complex	Yunnan	2021	[21]
*Colletotrichum ledongense*	*Colletotrichum gloeosporioides* complex	Hainan (Ledong)	2021	[22]

**Table 5:** Biological control mechanisms against rubber tree anthracnose.

Biocontrol Agent	Target Pathogen	Key Mechanisms	Efficacy	Reference
*Bacillus altitudinis* WR7	*Colletotrichum siamense*	Cell wall/membrane disruption, ROS induction, hydrolase/siderophore secretion, antimicrobial metabolites (lichenysin, fengycin, bacilysin), defense enzyme activation	82.36% inhibition (plate), 71.65% (pot)	[3]
*Bacillus velezensis* SF334	*Colletotrichum australisinense*, *Colletotrichum siamense*	Mycelial lysis through expansion and rupture, siderophore/protease/cellulase/pectinase/IAA secretion	79.67% prevention (*C. australisinense*)	[35]
*Bacillus subtilis* Czk1	*Colletotrichum* spp.	Lipopeptide production	Significant control (enhanced with Rootcon)	[35]
*Streptomyces badius* gz-8	*Colletotrichum gloeosporioides*	Dinactin production (EC_50_ = 2.55 μg/mL), inhibition of conidiogenesis and spore germination	72.5% (dual culture)	[46]
*Streptomyces deccanensis* QY-3	*Colletotrichum gloeosporioides*	Culture filtrate extract with antifungal compounds	Effective control	[47]
*Streptomyces* YQ-33, QZ-9, WZ-2	*Colletotrichum siamense*	Antagonistic activity	High antagonistic activity	[35]
*Curvularia eragrostidis*	*Colletotrichum tamarilloi*	Resistance induction (PAL activation)	10% less disease (preventive)	[43]
*Phialomyces macrosporus*	*Colletotrichum tamarilloi*	Resistance induction (PAL activation)	Positive curative effect	[43]
*Epicoccum dendrobii* SMEL1	*Colletotrichum* *gloeosporioides*	Antagonistic activity	Highly antagonistic	[35]

## Data Availability

All data generated or analyzed are included in the article.
